# Data Resource Profile: Nationwide registry data for high-throughput epidemiology and machine learning (FinRegistry)

**DOI:** 10.1093/ije/dyad091

**Published:** 2023-06-26

**Authors:** Essi Viippola, Sara Kuitunen, Rodosthenis S Rodosthenous, Andrius Vabalas, Tuomo Hartonen, Pekka Vartiainen, Joanne Demmler, Anna-Leena Vuorinen, Aoxing Liu, Aki S Havulinna, Vincent Llorens, Kira E Detrois, Feiyi Wang, Matteo Ferro, Antti Karvanen, Jakob German, Sakari Jukarainen, Javier Gracia-Tabuenca, Tero Hiekkalinna, Sami Koskelainen, Tuomo Kiiskinen, Elisa Lahtela, Susanna Lemmelä, Teemu Paajanen, Harri Siirtola, Mary Pat Reeve, Kati Kristiansson, Minna Brunfeldt, Mervi Aavikko, Finn Gen, Markus Perola, Andrea Ganna, FinnGen, Aarno Palotie, Mark Daly, Bridget Riley-Gills, Howard Jacob, Dirk Paul, Athena Matakidou, Adam Platt, Heiko Runz, Sally John, George Okafo, Nathan Lawless, Heli Salminen-Mankonen, Robert Plenge, Joseph Maranville, Mark McCarthy, Margaret G Ehm, Kirsi Auro, Simonne Longerich, Caroline Fox, Anders Mälarstig, Katherine Klinger, Clement Chatelain, Matthias Gossel, Karol Estrada, Robert Graham, Robert Yang, Chris ÓDonnell, Tomi P Mäkelä, Jaakko Kaprio, Petri Virolainen, Antti Hakanen, Terhi Kilpi, Jukka Partanen, Anne Pitkäranta, Taneli Raivio, Raisa Serpi, Tarja Laitinen, Veli-Matti Kosma, Jari Laukkanen, Marco Hautalahti, Outi Tuovila, Raimo Pakkanen, Jeffrey Waring, Bridget Riley-Gillis, Fedik Rahimov, Ioanna Tachmazidou, Chia-Yen Chen, Heiko Runz, Zhihao Ding, Marc Jung, Shameek Biswas, Rion Pendergrass, Margaret G Ehm, David Pulford, Neha Raghavan, Adriana Huertas-Vazquez, Jae-Hoon Sul, Anders Mälarstig, Xinli Hu, Åsa Hedman, Katherine Klinger, Robert Graham, Manuel Rivas, Dawn Waterworth, Nicole Renaud, Máen Obeidat, Samuli Ripatti, Johanna Schleutker, Markus Perola, Mikko Arvas, Olli Carpén, Reetta Hinttala, Johannes Kettunen, Arto Mannermaa, Katriina Aalto-Setälä, Mika Kähönen, Jari Laukkanen, Johanna Mäkelä, Reetta Kälviäinen, Valtteri Julkunen, Hilkka Soininen, Anne Remes, Mikko Hiltunen, Jukka Peltola, Minna Raivio, Pentti Tienari, Juha Rinne, Roosa Kallionpää, Juulia Partanen, Ali Abbasi, Adam Ziemann, Nizar Smaoui, Anne Lehtonen, Susan Eaton, Heiko Runz, Sanni Lahdenperä, Shameek Biswas, Natalie Bowers, Edmond Teng, Rion Pendergrass, Fanli Xu, David Pulford, Kirsi Auro, Laura Addis, John Eicher, Qingqin S Li, Karen He, Ekaterina Khramtsova, Neha Raghavan, Martti Färkkilä, Jukka Koskela, Sampsa Pikkarainen, Airi Jussila, Katri Kaukinen, Timo Blomster, Mikko Kiviniemi, Markku Voutilainen, Mark Daly, Ali Abbasi, Jeffrey Waring, Nizar Smaoui, Fedik Rahimov, Anne Lehtonen, Tim Lu, Natalie Bowers, Rion Pendergrass, Linda McCarthy, Amy Hart, Meijian Guan, Jason Miller, Kirsi Kalpala, Melissa Miller, Xinli Hu, Kari Eklund, Antti Palomäki, Pia Isomäki, Laura Pirilä, Oili Kaipiainen-Seppänen, Johanna Huhtakangas, Nina Mars, Ali Abbasi, Jeffrey Waring, Fedik Rahimov, Apinya Lertratanakul, Nizar Smaoui, Anne Lehtonen, Marla Hochfeld, Natalie Bowers, Rion Pendergrass, Jorge Esparza Gordillo, Kirsi Auro, Dawn Waterworth, Fabiana Farias, Kirsi Kalpala, Nan Bing, Xinli Hu, Tarja Laitinen, Margit Pelkonen, Paula Kauppi, Hannu Kankaanranta, Terttu Harju, Riitta Lahesmaa, Nizar Smaoui, Glenda Lassi, Susan Eaton, Hubert Chen, Rion Pendergrass, Natalie Bowers, Joanna Betts, Kirsi Auro, Rajashree Mishra, Majd Mouded, Debby Ngo, Teemu Niiranen, Felix Vaura, Veikko Salomaa, Kaj Metsärinne, Jenni Aittokallio, Mika Kähönen, Jussi Hernesniemi, Daniel Gordin, Juha Sinisalo, Marja-Riitta Taskinen, Tiinamaija Tuomi, Timo Hiltunen, Jari Laukkanen, Amanda Elliott, Mary Pat Reeve, Sanni Ruotsalainen, Benjamin Challis, Dirk Paul, Natalie Bowers, Rion Pendergrass, Audrey Chu, Kirsi Auro, Dermot Reilly, Mike Mendelson, Jaakko Parkkinen, Melissa Miller, Tuomo Meretoja, Heikki Joensuu, Olli Carpén, Johanna Mattson, Eveliina Salminen, Annika Auranen, Peeter Karihtala, Päivi Auvinen, Klaus Elenius, Johanna Schleutker, Esa Pitkänen, Nina Mars, Mark Daly, Relja Popovic, Jeffrey Waring, Bridget Riley-Gillis, Anne Lehtonen, Jennifer Schutzman, Natalie Bowers, Rion Pendergrass, Diptee Kulkarni, Kirsi Auro, Alessandro Porello, Andrey Loboda, Heli Lehtonen, Stefan McDonough, Sauli Vuoti, Kai Kaarniranta, Joni A Turunen, Terhi Ollila, Hannu Uusitalo, Juha Karjalainen, Esa Pitkänen, Mengzhen Liu, Heiko Runz, Stephanie Loomis, Erich Strauss, Natalie Bowers, Hao Chen, Rion Pendergrass, Kaisa Tasanen, Laura Huilaja, Katariina Hannula-Jouppi, Teea Salmi, Sirkku Peltonen, Leena Koulu, Nizar Smaoui, Fedik Rahimov, Anne Lehtonen, David Choy, Rion Pendergrass, Dawn Waterworth, Kirsi Kalpala, Ying Wu, Pirkko Pussinen, Aino Salminen, Tuula Salo, David Rice, Pekka Nieminen, Ulla Palotie, Maria Siponen, Liisa Suominen, Päivi Mäntylä, Ulvi Gursoy, Vuokko Anttonen, Kirsi Sipilä, Rion Pendergrass, Hannele Laivuori, Venla Kurra, Laura Kotaniemi-Talonen, Oskari Heikinheimo, Ilkka Kalliala, Lauri Aaltonen, Varpu Jokimaa, Johannes Kettunen, Marja Vääräsmäki, Outi Uimari, Laure Morin-Papunen, Maarit Niinimäki, Terhi Piltonen, Katja Kivinen, Elisabeth Widen, Taru Tukiainen, Mary Pat Reeve, Mark Daly, Niko Välimäki, Eija Laakkonen, Jaakko Tyrmi, Heidi Silven, Eeva Sliz, Riikka Arffman, Susanna Savukoski, Triin Laisk, Natalia Pujol, Mengzhen Liu, Bridget Riley-Gillis, Rion Pendergrass, Janet Kumar, Kirsi Auro, Iiris Hovatta, Chia-Yen Chen, Erkki Isometsä, Hanna Ollila, Jaana Suvisaari, Thomas Damm Als, Antti Mäkitie, Argyro Bizaki-Vallaskangas, Sanna Toppila-Salmi, Tytti Willberg, Elmo Saarentaus, Antti Aarnisalo, Eveliina Salminen, Elisa Rahikkala, Johannes Kettunen, Kristiina Aittomäki, Fredrik Åberg, Mitja Kurki, Samuli Ripatti, Mark Daly, Juha Karjalainen, Aki Havulinna, Juha Mehtonen, Priit Palta, Shabbeer Hassan, Pietro Della Briotta Parolo, Wei Zhou, Mutaamba Maasha, Shabbeer Hassan, Susanna Lemmelä, Aarno Palotie, Aoxing Liu, Arto Lehisto, Andrea Ganna, Vincent Llorens, Hannele Laivuori, Taru Tukiainen, Mary Pat Reeve, Henrike Heyne, Nina Mars, Joel Rämö, Elmo Saarentaus, Hanna Ollila, Rodos Rodosthenous, Satu Strausz, Tuula Palotie, Kimmo Palin, Javier Garcia-Tabuenca, Harri Siirtola, Tuomo Kiiskinen, Jiwoo Lee, Kristin Tsuo, Amanda Elliott, Kati Kristiansson, Mikko Arvas, Kati Hyvärinen, Jarmo Ritari, Olli Carpén, Johannes Kettunen, Katri Pylkäs, Eeva Sliz, Minna Karjalainen, Tuomo Mantere, Eeva Kangasniemi, Sami Heikkinen, Arto Mannermaa, Eija Laakkonen, Nina Pitkänen, Samuel Lessard, Clément Chatelain, Perttu Terho, Tiina Wahlfors, Jukka Partanen, Eero Punkka, Raisa Serpi, Sanna Siltanen, Veli-Matti Kosma, Teijo Kuopio, Anu Jalanko, Huei-Yi Shen, Risto Kajanne, Mervi Aavikko, Henna Palin, Malla-Maria Linna, Mitja Kurki, Juha Karjalainen, Pietro Della Briotta Parolo, Arto Lehisto, Juha Mehtonen, Wei Zhou, Masahiro Kanai, Mutaamba Maasha, Zhili Zheng, Hannele Laivuori, Aki Havulinna, Susanna Lemmelä, Tuomo Kiiskinen, L Elisa Lahtela, Mari Kaunisto, Elina Kilpeläinen, Timo P Sipilä, Oluwaseun Alexander Dada, Awaisa Ghazal, Anastasia Kytölä, Rigbe Weldatsadik, Sanni Ruotsalainen, Kati Donner, Timo P Sipilä, Anu Loukola, Päivi Laiho, Tuuli Sistonen, Essi Kaiharju, Markku Laukkanen, Elina Järvensivu, Sini Lähteenmäki, Lotta Männikkö, Regis Wong, Auli Toivola, Minna Brunfeldt, Hannele Mattsson, Kati Kristiansson, Susanna Lemmelä, Sami Koskelainen, Tero Hiekkalinna, Teemu Paajanen, Priit Palta, Kalle Pärn, Mart Kals, Shuang Luo, Tarja Laitinen, Mary Pat Reeve, Shanmukha Sampath Padmanabhuni, Marianna Niemi, Harri Siirtola, Javier Gracia-Tabuenca, Mika Helminen, Tiina Luukkaala, Iida Vähätalo, Jyrki Tammerluoto, Marco Hautalahti, Johanna Mäkelä, Sarah Smith, Tom Southerington, Petri Lehto, Markus Perola

**Affiliations:** Institute for Molecular Medicine Finland (FIMM), HiLIFE, University of Helsinki, Helsinki, Finland; Institute for Molecular Medicine Finland (FIMM), HiLIFE, University of Helsinki, Helsinki, Finland; Public Health and Welfare, Finnish Institute for Health and Welfare (THL), Helsinki, Finland; Institute for Molecular Medicine Finland (FIMM), HiLIFE, University of Helsinki, Helsinki, Finland; Institute for Molecular Medicine Finland (FIMM), HiLIFE, University of Helsinki, Helsinki, Finland; Institute for Molecular Medicine Finland (FIMM), HiLIFE, University of Helsinki, Helsinki, Finland; Institute for Molecular Medicine Finland (FIMM), HiLIFE, University of Helsinki, Helsinki, Finland; Institute for Molecular Medicine Finland (FIMM), HiLIFE, University of Helsinki, Helsinki, Finland; Institute for Molecular Medicine Finland (FIMM), HiLIFE, University of Helsinki, Helsinki, Finland; Institute for Molecular Medicine Finland (FIMM), HiLIFE, University of Helsinki, Helsinki, Finland; Eric and Wendy Schmidt Center, Broad Institute of MIT and Harvard, Cambridge, MA, USA; Institute for Molecular Medicine Finland (FIMM), HiLIFE, University of Helsinki, Helsinki, Finland; Public Health and Welfare, Finnish Institute for Health and Welfare (THL), Helsinki, Finland; Institute for Molecular Medicine Finland (FIMM), HiLIFE, University of Helsinki, Helsinki, Finland; Institute for Molecular Medicine Finland (FIMM), HiLIFE, University of Helsinki, Helsinki, Finland; Institute for Molecular Medicine Finland (FIMM), HiLIFE, University of Helsinki, Helsinki, Finland; Institute for Molecular Medicine Finland (FIMM), HiLIFE, University of Helsinki, Helsinki, Finland; Institute for Molecular Medicine Finland (FIMM), HiLIFE, University of Helsinki, Helsinki, Finland; Institute for Molecular Medicine Finland (FIMM), HiLIFE, University of Helsinki, Helsinki, Finland; Eric and Wendy Schmidt Center, Broad Institute of MIT and Harvard, Cambridge, MA, USA; Institute for Molecular Medicine Finland (FIMM), HiLIFE, University of Helsinki, Helsinki, Finland; Institute for Molecular Medicine Finland (FIMM), HiLIFE, University of Helsinki, Helsinki, Finland; TAUCHI Research Center, Tampere University, Tampere, Finland; Public Health and Welfare, Finnish Institute for Health and Welfare (THL), Helsinki, Finland; Public Health and Welfare, Finnish Institute for Health and Welfare (THL), Helsinki, Finland; Institute for Molecular Medicine Finland (FIMM), HiLIFE, University of Helsinki, Helsinki, Finland; Institute for Molecular Medicine Finland (FIMM), HiLIFE, University of Helsinki, Helsinki, Finland; Institute for Molecular Medicine Finland (FIMM), HiLIFE, University of Helsinki, Helsinki, Finland; Public Health and Welfare, Finnish Institute for Health and Welfare (THL), Helsinki, Finland; Public Health and Welfare, Finnish Institute for Health and Welfare (THL), Helsinki, Finland; TAUCHI Research Center, Tampere University, Tampere, Finland; Institute for Molecular Medicine Finland (FIMM), HiLIFE, University of Helsinki, Helsinki, Finland; Public Health and Welfare, Finnish Institute for Health and Welfare (THL), Helsinki, Finland; Public Health and Welfare, Finnish Institute for Health and Welfare (THL), Helsinki, Finland; Institute for Molecular Medicine Finland (FIMM), HiLIFE, University of Helsinki, Helsinki, Finland; Public Health and Welfare, Finnish Institute for Health and Welfare (THL), Helsinki, Finland; Institute for Molecular Medicine Finland (FIMM), HiLIFE, University of Helsinki, Helsinki, Finland; Analytic and Translational Genetics Unit, Department of Medicine, Massachusetts General Hospital, Boston, MA, USA

Key Features

- FinRegistry is a curated, nationwide, register-based data resource for developing statistical and machine learning models, performing high-throughput epidemiological analyses and deriving outcome-specific prediction models.
- FinRegistry data are collected across 19 registries covering public health care visits, health conditions, medications, vaccinations, laboratory responses, demographics, familial relations and socioeconomic variables, with decades of follow-up for most registries.
- FinRegistry includes everyone living in Finland on 1 January 2010, as well as their parents, spouses, children and siblings, comprising a sample size of approximately 7.2 million persons.
- FinRegistry data are mapped to more than 3000 clinical endpoints defined by leveraging multiple registers and clinical expertise as part of the FinnGen project. The Risteys web portal [https://risteys.finregistry.fi] enables exploration of clinical endpoint definitions, their links to international ontologies and the results of epidemiological analyses to gain insights into disease epidemiology in the Finnish population.
- Access to FinRegistry is granted via the Finnish Social and Health Data Permit Authority Findata, which provides a clear and transparent application process and delivers the pseudonymized data in a secure computing environment.

## Data resource basics

Nationwide health-related registry data provide comprehensive insights into population health and, combined with other data such as demographics, familial relations and socioeconomic data, enable the exploration of various dimensions of human behaviour and health. With the increasing size and variety of available data, advanced statistical and machine learning methods present novel possibilities for prediction and causal inference.^1–3^ At the same time, efforts to extract high-quality ‘phenotypes’ from registries, for example clinical endpoints, are needed to provide interpretable results. In this spirit, projects such as the CALIBER initiative have provided curated phenotype definitions based on UK’s primary and secondary health care data.^4^ Traditionally, the identification of risk factors and the creation of prediction models for diseases have been conducted using a targeted approach where a specific condition, risk factor or medication is studied for its association with a single disease. Several studies^4–10^ have shown the potential of data-driven approaches in examining the associations of a large number of risk factors and thousands of disease trajectories. These studies have been accompanied by an increasing trend towards making the results publicly available through web portals, to enable the re-use of the results by other researchers.

The FinRegistry research project [www.finregistry.fi] seeks to model the complex relationship between health and various risk factors by developing statistical and machine learning models using high-resolution longitudinal registry data. The project is a joint effort led by the Finnish Institute for Health and Welfare (THL) and the Institute for Molecular Medicine Finland (FIMM), University of Helsinki. Access to FinRegistry is granted via the Finnish Social and Health Data Permit Authority Findata, which provides a clear and transparent application process and delivers the data in a secure computing environment. No ethics approval is required but instead, Findata examines the data access requests and grants a fixed-term data permit for processing confidential materials containing personal data under the Act on the Secondary Use of Health and Social Data.^11^ FinRegistry data are collected, used and stored in accordance with the General Data Protection Regulation. FinRegistry is funded by the European Research Council under the European Union’s Horizon 2020 research and innovation programme.

FinRegistry data are collected across 19 registries covering the Finnish population’s public health care visits, health conditions, medications, vaccinations, laboratory responses, demographics, familial relations and socioeconomic variables. As in other Nordic countries, the data are collected in nationwide electronic registries.^12^ The earliest year of data collection varies by the registry, with the Finnish Cancer Registry being the oldest and dating back to 1953. Pseudonymized individual-level data from different registers can be linked together using pseudo-IDs that replace the unique personal identification number assigned to each individual residing in Finland, and familial relations allow the connection of individuals with their close relatives and their respective registry data. Furthermore, including geospatial data (geographical coordinates of the place of residence) enables the integration of open-access geographical data, such as the average environmental pollution of the area.

**Figure 1.**
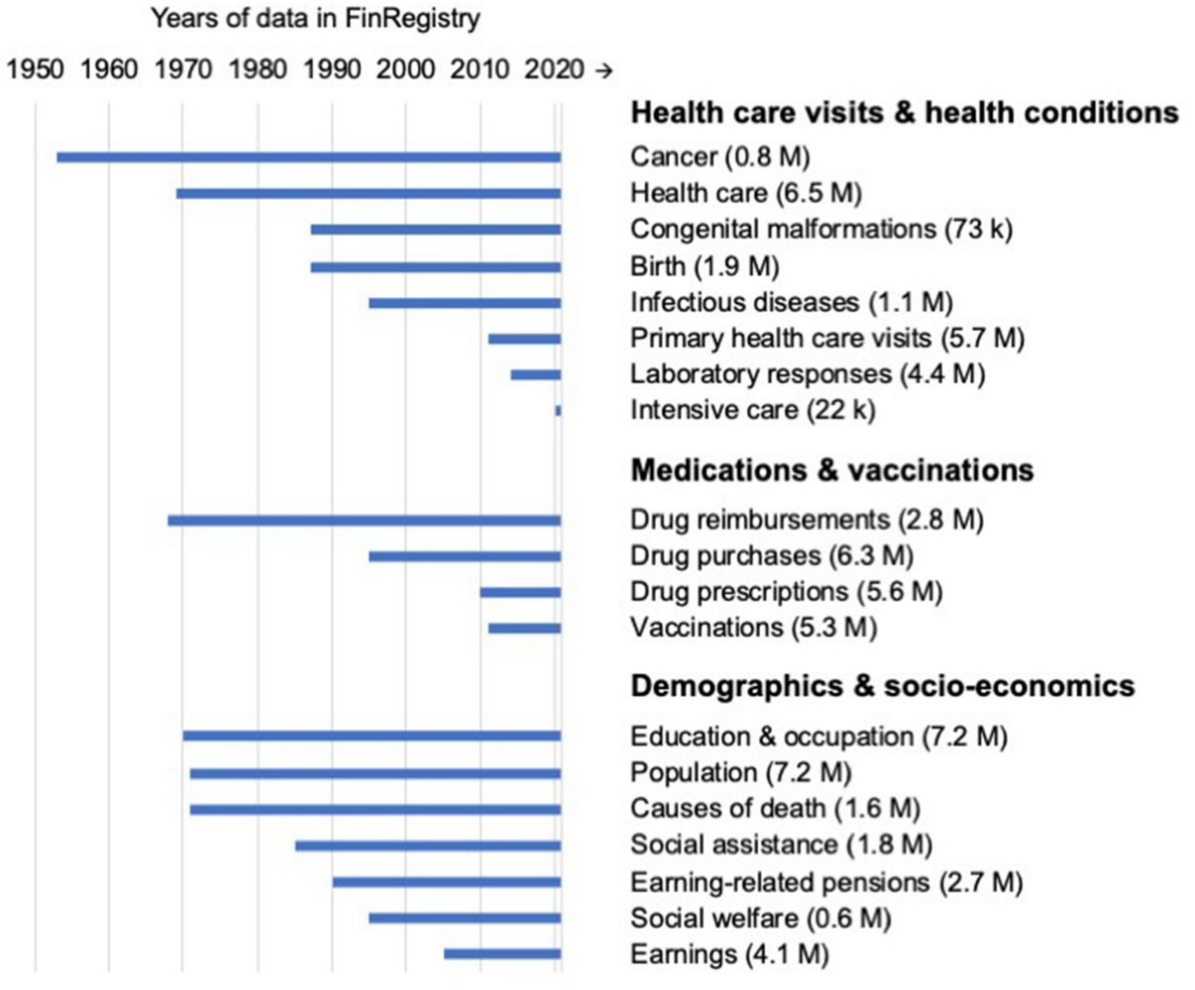
Finnish registries included in FinRegistry and the approximate number of unique individuals (in parentheses). Cancer, Finnish Cancer Registry; health care, Care Register for Health Care (Hilmo); congenital malformations, Register of Congenital Malformations; Birth, Medical Birth Register; infectious diseases, Finnish National Infectious Diseases Register; primary health care visits, Register of Primary Health Care Visits (AvoHilmo); laboratory responses, Kanta Laboratory Responses; intensive care, Intensive Care Registry; drug prescriptions, Kanta Prescription Centre and Prescription Archive; vaccinations, Finnish National Vaccination Register and Monitoring of the Vaccination Programme; population, Population Registry; Social assistance, Register of Social Assistance; Social welfare, Care Register for Social Welfare (Social Hilmo)

The study population in FinRegistry is fully representative of the Finnish population: FinRegistry covers individuals living in Finland on 1 January 2010 (FinRegistry index persons) as well as their parents, spouses, children and siblings (non-index relatives), with the exception of individuals excluded due to non-disclosure for personal safety reasons. To date, the data comprise 5 339 804 index persons and 1 826 612 non-index relatives, making up a total sample size of approximately 7.2 million individuals. The number of persons included and the years covered by each registry are presented in Figure 1, and more details are available in Supplementary Table S1 (available as Supplementary data at *IJE* online).

FinRegistry is a unique nationwide registry resource because of: (i) the scale and diversity of data linkage; (ii) extensive quality control, including the generation of curated health register-based clinical endpoints obtained by leveraging multiple registries and clinical expertise as part of the FinnGen project^13^; and (iii) access to descriptive statistics and high-throughput epidemiological analyses via the Risteys web portal [https://risteys.finregistry.fi]. Furthermore, we are planning to map FinRegistry data to the Observational Medical Outcomes Partnership Common Data Model (OMOP-CMD) as described in the Supplementary Materials (available as Supplementary data at *IJE* online).

## Data collected

### Data sources

FinRegistry data can be broadly categorized into the following partially overlapping categories: (i) health care visits and health conditions; (ii) medications and vaccinations; and (iii) demographics and socioeconomics. Registers included in each category and the years and numbers of persons covered are presented in Supplementary Table S1. A publicly available data dictionary is linked on the FinRegistry website [www.finregistry.fi/finnish-registry-data] and the code for data preprocessing is available on GitHub.^14^

*Health care visits and health conditions* comprise extensive data resulting from patient contacts with primary (since 2011 in FinRegistry), secondary (since 1969) and intensive care (since 2020), and cover details on the health care specialty. Additional information is available on psychiatric patients and those with demanding heart diseases (since 1994). Primary and home care information has been collected since 2011 and was recently extended to include data from private health care. Private service providers account for approximately a quarter of Finland’s social and health services.^15^ Laboratory results collected as part of public and private health care (e.g. blood glucose levels, blood cell counts and liver enzymes) are available since 2014. Disease-specific information on cancer (since 1953), microbiologically-confirmed infectious diseases (since 1995), including COVID-19, and congenital malformations (since 1987) are included in separate registers. Detailed information on the mother’s pregnancy, pregnancy-related risk factors, the delivery, the neonate’s information and neonatal conditions are also collected in the Medical Birth Register (since 1987).

*Medications and vaccinations* cover the purchase (since 1995) of reimbursable medicines, all electronic prescriptions and their delivery records made in pharmacies (since 2010), as well as the vaccinations given in public health care (since 2011). The medication-related registries include information on pharmaceutical attributes, such as the Anatomical Therapeutic Chemical (ATC) classification code,^16^ package size, formulation, dosage and cost. The underlying health indications for prescribing certain medications are reported in text format during the prescription, but this information is incomplete for some entries. Of note is that certain health conditions for which medications are eligible for reimbursement, such as type 2 diabetes, are recorded in the Drug Reimbursements register and represent a high-quality source to identify disease diagnoses as early as 1968. Vaccination information contains, among others, the administration of COVID-19 vaccines.

*Demographic information and socioeconomics data* include sex, date of birth and death, familial relations, marriage history and the longitudinal coordinates of the place of residence, including immigration and emigration dates, as well as information on education, employment, labour income, pensions and social assistance. A multigenerational register includes familial relations for first-degree relatives (mother, father, children and siblings) of the FinRegistry index persons. Based on first-degree relations, it is possible to construct a population-wide pedigree linking more distant relatives.^17^ Living history (since 1971) is used to link open-sourced geocoded data, such as the degree of urbanicity, the number of vacant housing units and the average amount of alcohol consumed. Marriage history (since 1971) includes marital status and its start and end date but does not include cohabiting couples. The date and causes of death provide valuable health information and have been recorded since 1971. Education, including the level and field of education, employment information, including the job profession, and the socioeconomic status, were initially collected via census in 1970, 1975 and 1985 and are available yearly since 1987. Information about earning-related pensions, pension rehabilitation and reason for incapacity to work (since 1990), income from labour (since 2005), including unpaid periods such as long-term sickness leaves and parental leaves, are available in two separate registers obtained from the Finnish Centre of Pensions. Financial support from the government has been recorded in the Registry of Social Assistance since 1985. Information about social welfare, including clients of institutional care and residential services, has been included in the Care Register for Social Welfare since 1995.

### Clinical endpoints and Risteys web portal

Clinical endpoints are indicators of certain medical conditions, e.g. an acute event such as myocardial infarction, or the onset of a chronic disease, such as type 1 diabetes. Clinical endpoints have been defined as part of the FinnGen project,^13^ a large-scale academic/industrial research collaboration aiming to collect and analyse genomic and health data from 500 000 Finnish biobank participants. Multiple registers (Care Register for Health Care, Register of Primary Health Care Visits, Causes of Death, Drug Purchases, Drug Reimbursements and Finnish Cancer Registry) have been used for defining the endpoints. The registries cover almost half a century of data during which, for example, the Finnish-specific International Classification of Diseases (ICD) versions 8 to 10 have been used, requiring harmonization of the data and the endpoint definitions. Currently, 3177 clinical endpoints have been defined in collaboration with clinical working groups with experience in using diagnostic codes in clinical practice. This phenotype library follows the ICD-10 hierarchy, covering the 21 chapters of diseases in ICD-10, with minor changes made in the hierarchy when ICD-8 and ICD-9 codes are necessary or when an endpoint is of specific interest. To make the clinical endpoints comparable with other international efforts, we have matched them with disease ontology coding systems Disease Ontology Identifier (DOID), Experimental Factor Ontology (EFO) and Medical Subject Headings (MeSH), as further described in the Supplementary Materials. Clinical endpoint definitions are available online.^18^

Risteys [https://risteys.finregistry.fi] is a publicly available web portal that enables exploration of clinical endpoints interactively. Risteys is the go-to place to gain insights into disease epidemiology in the Finnish population. The portal provides information on endpoint definitions and descriptive statistics in FinRegistry and FinnGen, including distributions of age and year at the first event, cumulative incidence estimates and mortality statistics. Risteys is constantly expanding to include results from high-throughput analyses performed in FinRegistry. The Risteys source code is available on GitHub^19^ and more information is presented in the Supplementary Materials.

## Data resource use

FinRegistry is a curated, nationwide, register-based data resource for developing statistical and machine learning models, performing high-throughput epidemiological analyses and deriving outcome-specific prediction models. For example, benefiting from the rich longitudinal health and multigeneration registers, we have used FinRegistry data to comprehensively assess the role and relative importance of 414 diseases in childlessness over the entire reproductive lifespan of both men and women.^20^ We have also implemented a machine learning approach to examine the association between 2890 health, socioeconomic, familial and demographic factors with the uptake of the first COVID-19 vaccination dose.^21^ Last, we have implemented the Risteys portal to enable exploration of clinical endpoints and the results of high-throughput epidemiological analyses, such as mortality statistics, as described below.

**Figure 2.**
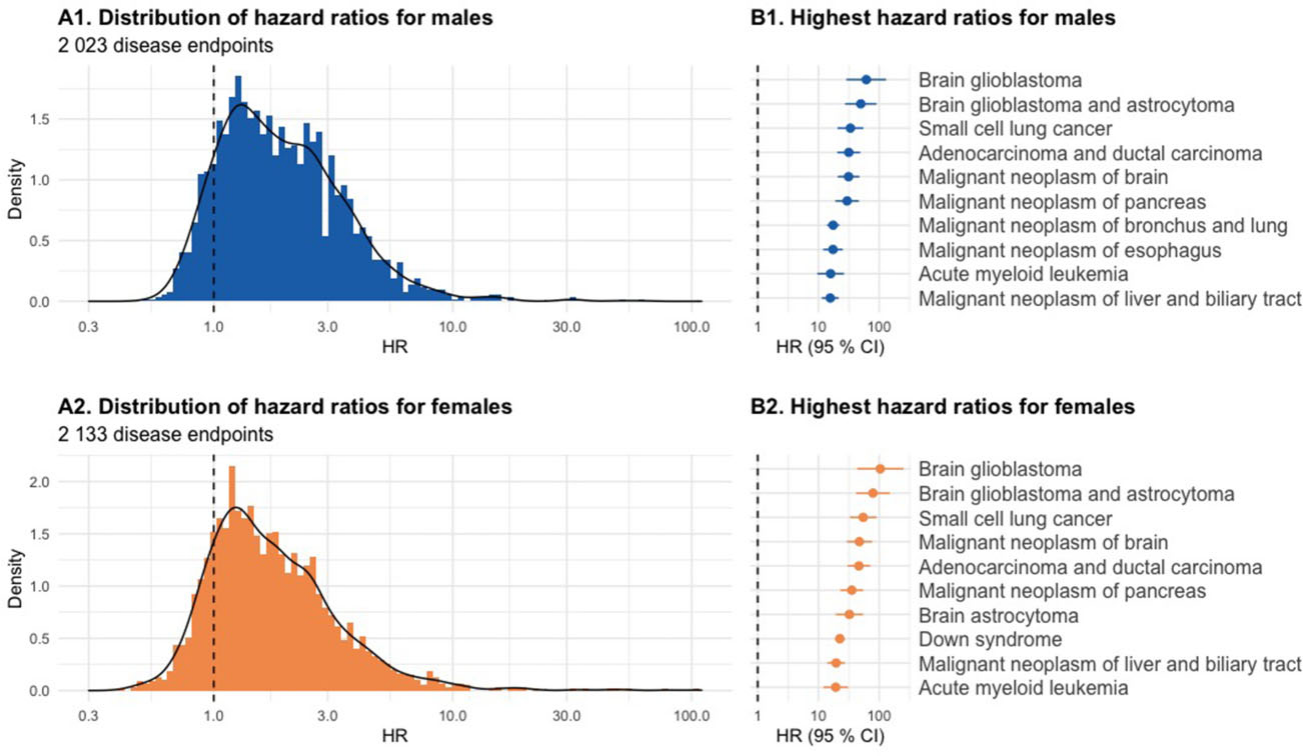
Distributions of hazard ratios for associations between each clinical endpoint and death (panels A1 and A2) and the highest hazard ratios (panels B1 and B2) for males and females. HR, hazard ratio; CI, confidence interval

High-throughput mortality analysis was applied to study the association between each clinical endpoint and death and to estimate the mortality risk associated with the clinical endpoints in the Finnish population. We used the Cox proportional hazards model^22^ with age as a time scale to estimate the hazard ratios (HR) between all clinical endpoints with sufficient data (2023 clinical endpoints for males and 2133 for females) and death. The eligible sample comprised 6 465 910 persons (50.59 % of whom were women) and 119 472 956 person-years, and the median follow-up was 22.0 [interquartile range (IQR) 16.7 to 22.0] years. The distribution of the obtained HRs, along with the highest HR for both sexes, are presented in Figure 2. The median HR was 1.8 (IQR 1.2 to 2.8) for males and 1.6 (IQR 1.2 to 2.5) for females. For both sexes, the highest HRs were obtained for brain glioblastoma [HR 61.3, 95 % confidence interval (28.9, 130.0) for males and 104.0 (43.3, 250.0) for females], brain glioblastoma and astrocytoma [49.8 (27.5, 90.2) for males and 79.0 (41.8, 149.0) for females] and small cell lung cancer [33.6 (20.6, 54.8) for males and 54.5 (32.9, 90.3) for females]. The results, including HRs and mortality risks, can be explored via the Risteys portal [https://risteys.finregistry.fi], and more information on the methods is available in the Supplementary Materials.

Ongoing projects using FinRegistry data focus on developing both traditional prediction models and novel machine/deep learning-based approaches. For example, we are currently developing a clinical prediction model to assess the infant’s risk of severe respiratory syncytial virus-caused disease which aims at helping the administration of novel immunoprophylaxis methods against the disease.^23^ Additionally, we are exploring methods to generate latent representations, or embeddings, across all FinRegistry data. These latent representations will help reduce the data dimensionality while identifying the major axes of variation in the underlying data. We are further leveraging the extensive population-scale pedigree by using Graph Neural Network to improve risk prediction. Finally, we are expanding the Risteys content by including additional high-throughput epidemiological analyses, such as disease-to-disease survival analyses.

## Strengths and weaknesses

FinRegistry data represent the entire population of Finland, allowing researchers to conduct large-scale cohort studies with a relatively low risk of selection bias and to study health events with higher statistical power. Finnish national registers include decades of data, with nearly a third of the registries covering half a century. For example, FinRegistry data have almost complete coverage of all major health-related events due to the inclusion of all treatments of severe and acute illnesses, emergency room visits, inpatient hospitalizations and major surgical operations carried out in the secondary health care of the public sector. The sociodemographic, social care and population registries similarly have virtually full coverage. The long follow-up is valuable when studying diseases with a long latent period between the exposure and the disease onset or in family-based studies requiring long follow-up periods for two or more generations. FinRegistry further combines the breadth of health data with a wide range of other information, including longitudinal data of the familial relations and the geographical coordinates of the place of residence, which in turn enables analysis of disease trajectories within families and linkage of the data to external datasets of the living environment. Finally, translating health data into clinical endpoints built on information obtained from multiple registries enhances the clinical relevance of the results and improves reproducibility across different health care systems.

As the data included in FinRegistry were not primarily collected for research, some clinically relevant variables are missing and the data do not cover all aspects of health care. Primary care-related data have been collected since 2011, with private health care being included only during recent years, and therefore the data coverage for conditions diagnosed and managed at the primary care level is limited. For instance, the Vaccination Register well covers COVID-19 vaccinations but not influenza vaccinations, as many of them are given in the private sector or occupational health care. Moreover, medications administered during hospital treatment (e.g. intravenous drugs) and non-prescription medications bought at pharmacies are not included. Primary care data include lifestyle and other modifiable risk factors, such as body mass index and smoking status, but a significant amount of data is missing. Such data are likely more often recorded if concerns are raised, resulting in lower coverage among healthy individuals. Despite the limitations above, FinRegistry provides unique, nationwide, integrated data covering various dimensions of human behaviour and health.

## Data resource access

Access to FinRegistry data can be obtained by submitting a data permit application for individual-level data to the Finnish Social and Health Data Permit Authority Findata [https://asiointi.findata.fi]. Data obtained through Findata covers all registries included in FinRegistry apart from education, job occupation and socioeconomic status, which can be obtained from Statistics Finland [https://lupa.stat.fi]. Requests are evaluated on a case-by-case basis. Once approved, the data are sent to a secure computing environment and can be accessed within the European Economic Area and countries with an adequacy decision from the European Commission. Detailed instructions for the Findata access process are available at [https://findata.fi/en/data]. Data dictionaries for FinRegistry are publicly available on the FinRegistry website [www.finregistry.fi/finnish-registry-data]. For more information, please contact Findata [info@findata.fi] or Dr Andrea Ganna [andrea.ganna@helsinki.fi].

## Ethics approval

FinRegistry is a collaboration project of the Finnish Institute for Health and Welfare (THL) and the Data Science Genetic Epidemiology research group at the Institute for Molecular Medicine Finland (FIMM), University of Helsinki. The FinRegistry project has received approvals for data access from THL (THL/1776/6.02.00/2019 and subsequent amendments), Digital and Population Data Services Agency (VRK/5722/2019–2), Finnish Center for Pension (ETK/SUTI 22003) and Statistics Finland (TK-53–1451-19). The FinRegistry project has received IRB approval from THL (Kokous 7/2019).

## Supplementary Material

dyad091_Supplementary_Data

## Data Availability

See Data Resource Access, above.
