# Supplementary material for: Data Resource Profile: Nationwide registry data for high-throughput epidemiology and machine learning (FinRegistry)
Source: Int J Epidemiol. 2023 Jun 26;52(4):e195–200. doi: 10.1093/ije/dyad091 (PMC10396416; doi:10.1093/ije/dyad091)
Supplement: dyad091_Supplementary_Data [file dyad091_supplementary_data.zip › dyad091_Supplementary_Data/ije-2023-02-0185-File004.docx]

# Supplementary materials

Supplementary Table S1. Registries included in FinRegistry, years covered, and the number of persons in FinRegistry.

| **Category** | **Registry and controller** | **Years in FinRegistry** | **Persons in FinRegistry** |
| --- | --- | --- | --- |
| Health care visits and health conditions | Finnish Cancer Registry, THL [1] | 1953– | 842 596 |
|  | Care Register for Health Care (Hilmo), THL [2] | 1969– (outpatient data since 1998) | 6 401 445 |
|  | Register of Congenital Malformations, THL [3] | 1987– | 73 359 |
|  | Medical Birth Register, THL [4] | 1987– | 1 913 226 |
|  | Finnish National Infectious Diseases Register, THL [5] | 1995– | 1 118 353 |
|  | Register of Primary Health Care Visits (AvoHilmo), THL [6] | 2011– | 5 678 354 |
|  | Kanta Laboratory Responses, Kela [7] | 2014– | 4 414 395 |
|  | Intensive Care Registry, FICC [8] | 2020– | 22 712 |
| Medications & vaccinations | Drug Reimbursements, Kela | 1968– | 2 762 185 |
|  | Drug Purchases, Kela | 1995– | 6 297 688 |
|  | Kanta Prescription Centre and Prescription Archive, Kela [9, 10] | 2010– | 5 577 137 |
|  | Finnish National Vaccination Register and Monitoring of the Vaccination Programme, THL [11] | 2011– | 5 276 014 |
| Demographics & socio-economics | Education, Job Occupation, and Socio-economic status, SF | 1970, 1975, 1985, 1987– | 7 166 196 |
|  | Population Registry, DVV [12] | 1971– | 7 166 196 |
|  | Causes of Death, SF [13] | 1971– | 1 614 295 |
|  | Register of Social Assistance, THL [14] | 1985– | 1 797 788 |
|  | Earning-related Pensions, FCP [15] | 1990– | 2 653 072 |
|  | Care Register for Social Welfare (Social Hilmo), THL [16] | 1995– | 580 422 |
|  | Earnings, FCP [17] | 2005– | 4 128 404 |

*THL, Finnish Institute for Health and Welfare; FICC, Finnish Intensive Care Consortium; Kela, The Social Insurance Institution; SF, Statistics Finland; DVV, Digital and Population Data Services Agency; FCP, Finnish Centre for Pensions*

## Risteys portal

Risteys (<https://risteys.finregistry.fi>) is an interactive web portal that enables users to explore clinical endpoints in the populations of FinRegistry and FinnGen. Risteys is based on FinnGen clinical endpoints [18], indicators of medical conditions defined by leveraging multiple registries and clinical expertise, and provides information on the clinical endpoint definitions and results of high-throughput epidemiological analyses.

The Risteys pipeline is outlined in **Supplementary Figure S1**. Clinical endpoint data, i.e. the age at the first event of each clinical endpoint, are generated in FinRegistry and FinnGen based on clinical endpoint definitions and combined with demographic data such as date of birth, date of death, and sex. Statistical analyses include summaries of the disease prevalence, incidence, and age at onset in both datasets and mortality analysis conducted in FinRegistry. The results of the analyses and the clinical endpoint definitions are transferred to a cloud-based database and visualised on the Risteys web portal. The contents of the Risteys portal include endpoint definitions and their links to international ontologies, as well as the results of the statistical analyses for all clinical endpoints with sufficient data.


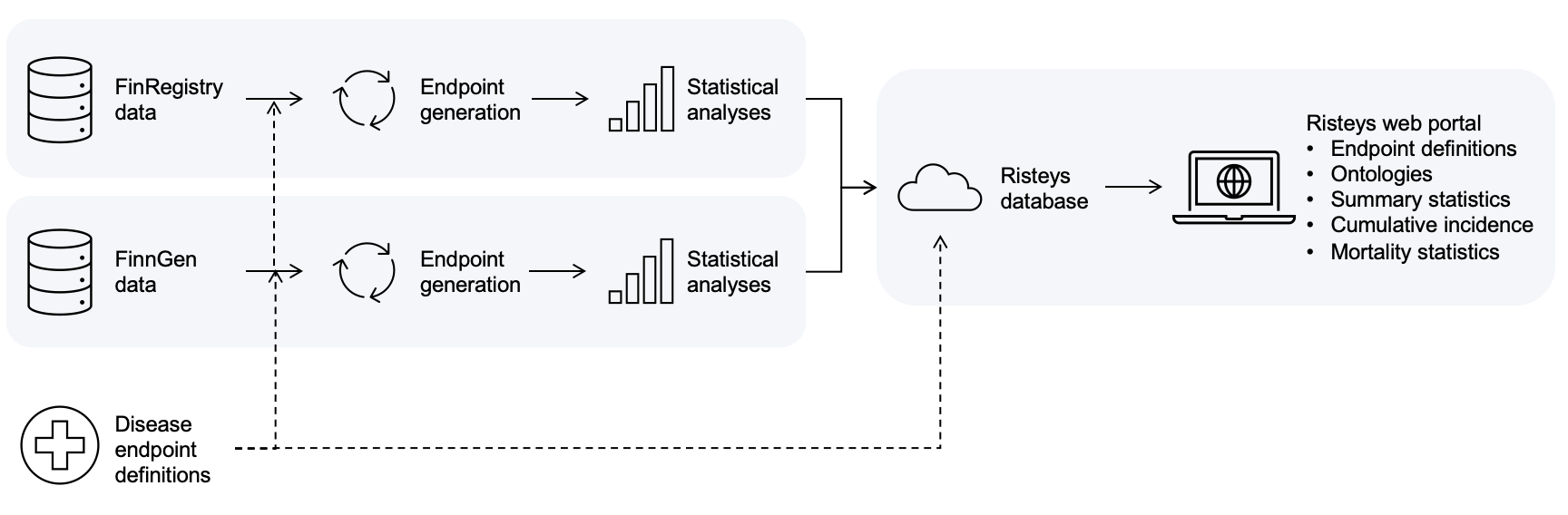


Supplementary Figure S1. Risteys pipeline.

Clinical endpoint definitions presented on Risteys show the steps for constructing each clinical endpoint and the number of persons included after each step. UpSet plots [19] are shown to describe further how the registry data contributes to each clinical endpoint. Clinical endpoints are matched with international ontologies using an automated algorithm followed by manual curation. Clinical endpoints are first linked to DOID and MeSH codes using a hierarchical algorithm: 1) clinical endpoint ICD-10 codes are matched to DOID ICD-10-CM codes, 2) clinical endpoint names are matched to DOID names and synonyms, 3) clinical endpoints are matched to MeSH codes and converted to DOID, 4) clinical endpoint ICD-10 codes are matched one step up in the ICD-10 hierarchy, 5) clinical endpoint names are matched with DOID codes using the Ratcliff/Obershelp pattern matching accepting similarity > 0.69. The resulting DOID and MeSH codes are mapped to EFO when available. Next, clinical endpoints are linked to EFO codes using the fuzzy matching algorithm OnToma [20] and the ontology annotations for clinical endpoints are available on the Open Targets portal (<https://www.opentargets.org>). Finally, clinical endpoints with discordant EFO annotations between OnToma, Open Targets, and the existing mappings are manually checked and corrected. The resulting DOID, EFO, and MeSH codes are linked to each endpoint on Risteys when available.

The key figures and the distributions for age and year at the first event are included in the summary statistics. Summary statistics are presented for FinRegistry and FinnGen, and key figures are additionally included for the subsample of FinRegistry index persons. Key figures include the number of individuals with the endpoint of interest, unadjusted period prevalence over the registry coverage, i.e. the case count divided by the cohort size, and the median age at the first event. Aggregate data of the age and year at the first event are presented as distributions. To present an overview of all available data, no follow-up period is applied at this stage.

The cumulative incidence function (CIF) shows the incidence of an endpoint by age and sex and is estimated using the Aalen-Johansen estimator [21] in a competing-risks framework where death is treated as a competing event. We use age as a timescale to obtain CIF estimates by age bin. The follow-up period starts on January 1, 1998, or birth, whichever occurs later, and ends on December 31, 2019, or death. The eligible sample includes all persons in FinRegistry and FinnGen who were born before the end of the follow-up and are either not dead or died during the follow-up period. Persons with missing sex are excluded, and only clinical endpoints occurring during the follow-up period are considered. In FinRegistry, the eligible sample comprised 6 465 910 persons (50.59 % of whom were women) and 119 472 956 person-years. The median follow-up was 22.0 (IQR 16.7 to 22.0) years. All or at most 10 000 cases and 1.5 non-cases per case are sampled for each endpoint, and the subjects are weighted by the inverse of the sampling probability to account for the sampling design. CIF is only estimated for endpoints with at least 50 cases and controls, and age bins with less than five persons are omitted due to the sensitive nature of the data. The CIF is estimated for all clinical endpoints with sufficient data, yielding 4 539 sex-stratified CIF estimates for 2 466 endpoints.

The association between the clinical endpoints and death is estimated using the Cox proportional hazards model [22] using age as a timescale. The same follow-up period and eligibility criteria are applied as in the CIF model. For each endpoint, we apply endpoint-stratified sampling to obtain a sufficient number of diseased cases (individuals who died during the follow-up) and controls. Sampling is carried out for each sex independently. We sample all or at most 5 000 individuals with and without a clinical endpoint each; similarly, 1.5 controls per case are sampled among the non-cases in both categories. For the diseased persons, the time before the disease onset is included as non-diseased time, and persons with less than 30 days between the disease onset and death are considered non-diseased. Samples with less than 50 persons are not included in further analysis. The Cox proportional hazards model is fit independently for each clinical endpoint and sex. We used age as a timescale to model the age-varying risk of death and included birth year as a covariate to account for the calendar effects. Disease status is further included as a covariate to estimate the hazard ratios averaged over the timescale. Each model is weighted by the inverse of the sampling probability. Hazard ratios are obtained for each endpoint by sex, yielding 4 156 hazard ratios, and the hazard ratios and cumulative baseline hazards were further used to predict 1, 5, 10, 15, and 20-year mortality risks of each endpoint for a given age using the year 2022 subtracted by age as the birth year. The 1-year absolute risks of a disease with a hazard ratio of approximately one were compared with the mortality statistics by Statistics Finland [23] to ensure the baseline hazards have been correctly estimated.

The data pipeline is implemented using Python version 3.9.7 and multiprocessing is applied due to the size of the data and the number of models fitted. The survival analysis models for the CIF and mortality analysis are implemented using Lifelines version 0.26.4 [24, 25]. FinRegistry and FinnGen data are processed in their respective secure computing environments using a harmonised data processing pipeline. The results of the statistical analyses are stored on a SQL database on the Google Cloud Platform. The Risteys web portal is implemented using Elixir and JavaScript. The source code for the harmonised data pipeline and the Risteys web portal is available on GitHub (MIT license) [26].

## OMOP Common Data Model

The Observational Medical Outcomes Partnership Common Data Model (OMOP-CDM) is a standard for the harmonisation of observational data governed by the Observational Health Data Sciences and Informatics (OHDSI) open community. The OMOP-CDM provides a standardised vocabulary of medical terms, database structure, and software tools to facilitate analysis. The main purpose of OHDSI is to conduct federate analysis. Typically, a research question is translated into analytic code, ran locally in multiple OMOP-CDM databases, and the aggregated summary results are collated centrally. However, OHDSI’s open-source software can also be used in local analyses, saving considerable time in tooling development and testing [27]. The OMOP-CDM network is made of more than a hundred databases and covers 600 million unique patients across the world [27]. In Europe, the European Medicines Agency has recently chosen OMOP-CDM to build a federated network to answer pharmacoepidemiological questions [28]. In Finland, the FinnGen project and the university hospitals of Helsinki, Turku, and Tampere have converted part of their medical records into OMOP-CDM.

Transforming FinRegistry data to the OMOP-CDM brings several advantages at local, national, and international levels. At the local level, FinRegistry users will be able to use the existing OHDSI software tools for phenotype definition and characterisation, population-level effect estimation, and patient-level prediction [29]. At the national level, it will allow for easy transportability of phenotype definition and machine learning models between databases. For example, a probabilistic phenotype trained in FinRegistry’s OMOP-CDM could be used to capture subjects in FinnGen’s OMOP-CDM database to be later used in GWAS analysis. At the international level, FinRegistry will be able to participate as a data partner in OHDSI federated studies or use other databases in the network to validate analysis design at FinRegistry.

## Supplementary references

1. Data Resources Catalogue. Finnish Cancer Registry. <https://aineistokatalogi.fi/catalog/studies/21085403-7be8-4f93-bf05-231518c642a0> (January 2023, date last accessed).
2. Finnish Institute for Health and Welfare. Care Register for Health Care. <https://thl.fi/en/web/thlfi-en/statistics-and-data/data-and-services/register-descriptions/care-register-for-health-care> (January 2023, date last accessed).
3. Finnish Institute for Health and Welfare. Register of Congenital Malformations. <https://thl.fi/en/web/thlfi-en/statistics-and-data/data-and-services/register-descriptions/register-of-congenital-malformations> (January 2023, date last accessed).
4. Finnish Institute for Health and Welfare. Medical Birth Register. <https://thl.fi/en/web/thlfi-en/statistics-and-data/data-and-services/register-descriptions/newborns> (January 2023, date last accessed).
5. Finnish Institute for Health and Welfare. Finnish National Infectious Diseases Register. <https://thl.fi/en/web/infectious-diseases-and-vaccinations/surveillance-and-registers/finnish-national-infectious-diseases-register> (January 2023, date last accessed).
6. Data Resources Catalogue. Register of Primary Health Care Visits 2011- (AvoHilmo). <https://aineistokatalogi.fi/catalog/studies/7567e45d-72b7-428b-be9e-510440336edf/datasets/25ff4fc4-6166-4e6c-b6b8-e9c4c1a2cc7d> (January 2023, date last accessed).
7. Data Resources Catalogue. Laboratoriovastaukset. <https://aineistokatalogi.fi/catalog/studies/3e9d936e-ee2a-4e0e-9344-fb2c85b94e0c/datasets/b9df6cfe-d9ef-48da-aca2-d5d6e345ceed> (January 2023, date last accessed).
8. Reinikainen M, Karlsson M, Okkonen M, Linko R. Suomen tehohoitokonsortion laatutietokannan hyödyntäminen tieteellisessä tutkimuksessa. *Finnanest* 2012; **45**(4): 324-329.
9. Data Resources Catalogue. Lääkemääräykset (Kanta - Reseptikeskus). <https://aineistokatalogi.fi/catalog/studies/ff551f4e-6842-4732-962d-74499a339c46/datasets/aaa2dd17-729a-455f-8e96-82f66a43a746> (January 2023, date last accessed).
10. Data Resources Catalogue. Lääketoimitukset (Kanta - Reseptikeskus). <https://aineistokatalogi.fi/catalog/studies/ff551f4e-6842-4732-962d-74499a339c46/datasets/0d173c62-57ff-4dfe-8bdd-1065e63217b9> (January 2023, date last accessed).
11. Finnish Institute for Health and Welfare. Finnish National Vaccination Register and Monitoring of the Vaccination Programme. <https://thl.fi/en/web/infectious-diseases-and-vaccinations/surveillance-and-registers/finnish-national-vaccination-register-and-monitoring-of-the-vaccination-programme> (January 2023, date last accessed).
12. Digital and Population Data Services Agency. Population Information System. <https://dvv.fi/en/population-information-system> (January 2023, date last accessed).
13. Data Resources Catalogue. Causes of Death - Research Data. <https://aineistokatalogi.fi/catalog/studies/778c33bf-aceb-423f-89d9-e5abb5a0585c> (January 2023, date last accessed).
14. Finnish Institute for Health and Welfare. Register of Social Assistance. <https://thl.fi/en/web/thlfi-en/statistics-and-data/data-and-services/register-descriptions/social-assistance> (January 2023, date last accessed).
15. Data Resources Catalogue. Pensions Register. <https://aineistokatalogi.fi/catalog/studies/50b7d99a-3e5e-495c-874b-7622a0c897c3> (January 2023, date last accessed).
16. Data Resources Catalogue. Care Register for Social Welfare 1995- (Sosiaalihilmo). <https://aineistokatalogi.fi/catalog/studies/7567e45d-72b7-428b-be9e-510440336edf/datasets/abcc2578-1a24-41e0-bce9-2c214538b087> (January 2023, date last accessed).
17. Data Resources Catalogue. Pension-insured Employment in Earnings Register. <https://aineistokatalogi.fi/catalog/studies/a30b0998-53e8-4846-b31f-eb825ce463c6> (January 2023, date last accessed).
18. FinnGen. Clinical endpoints. <https://www.finngen.fi/en/researchers/clinical-endpoints> (February 2023, date last accessed).
19. Lex A, Gehlenborg N, Strobelt H, Vuillemot R, Pfister H. UpSet: Visualization of Intersecting Sets. *IEEE Trans Vis Comput Graph*. 2014;**20**(12):1983–92.
20. Open Targets. OnToma. GitHub, <https://github.com/opentargets/OnToma>, 2022.
21. Aalen OO, Johansen S. An Empirical Transition Matrix for Non-Homogeneous Markov Chains Based on Censored Observations. *Scand J Stat.* 1978;**5**(3):141–50.
22. Cox DR. Regression Models and Life-Tables. *J R Stat Soc Series B Stat Methodol.* 1972;**34**(2):187–202.
23. Statistics Finland. Official Statistics of Finland (OSF): Deaths. <http://www.stat.fi/til/kuol/index_en.html> (October 2022, date last accessed).
24. Davidson-Pilon C. lifelines. Zenodo, doi: 10.5281/zenodo.5745573, 2021.
25. Davidson-Pilon C. lifelines: survival analysis in Python. *JOSS* 2019;**4**(40):1317.
26. Llorens V, Kuitunen S, Viippola E, Reeve MP, Ganna A. Risteys, version 2.0.0. GitHub, <https://github.com/dsgelab/risteys/tree/v2.0.0>, 2022.
27. Hripcsak G, Schuemie MJ, Madigan D, Ryan PB, Suchard MA. Drawing Reproducible Conclusions from Observational Clinical Data with OHDSI. *Yearb Med Inform* 2021;**30**(1):283–289.
28. European Medicines Agency. Data Analysis and Real World Interrogation Network (DARWIN EU). <https://www.ema.europa.eu/en/about-us/how-we-work/big-data/data-analysis-real-world-interrogation-network-darwin-eu> (October 2022, date last accessed).
29. Observational Health Data Sciences and Informatics. GitHub, <https://github.com/OHDSI/>.
